# Detection of Thrombin Based on Fluorescence Energy Transfer between Semiconducting Polymer Dots and BHQ-Labelled Aptamers

**DOI:** 10.3390/s18020589

**Published:** 2018-02-14

**Authors:** Yizhang Liu, Xuekai Jiang, Wenfeng Cao, Junyong Sun, Feng Gao

**Affiliations:** 1 Department of Food and Environmental Engineering, Vocational and Technical College, Chuzhou 239001, China; 2 Laboratory of Functionalized Molecular Solids, Ministry of Education, Anhui Key Laboratory of Chemo/Biosensing, Laboratory of Optical Probes and Bioelectrocatalysis (LOPAB), College of Chemistry and Materials Science, Anhui Normal University, Wuhu 241000, China; jiangxuekai@ahnu.edu.cn (X.J.); wfcxi17@mail.ahnu.edu.cn (W.C.); sunjy228@mail.ahnu.edu.cn (J.S.)

**Keywords:** semiconducting polymer dots, thrombin, aptamer, fluorescence resonance energy trans

## Abstract

Carboxyl-functionalized semiconducting polymer dots (Pdots) were synthesized as an energy donor by the nanoprecipitation method. A black hole quenching dye (BHQ-labelled thrombin aptamers) was used as the energy acceptor, and fluorescence resonance energy transfer between the aptamers and Pdots was used for fluorescence quenching of the Pdots. The addition of thrombin restored the fluorescence intensity. Under the optimized experimental conditions, the fluorescence of the system was restored to the maximum when the concentration of thrombin reached 130 nM, with a linear range of 0–50 nM (R^2^ = 0.990) and a detection limit of 0.33 nM. This sensor was less disturbed by impurities, showing good specificity and signal response to thrombin, with good application in actual samples. The detection of human serum showed good linearity in the range of 0–30 nM (R^2^ = 0.997), with a detection limit of 0.56 nM and a recovery rate of 96.2–104.1%, indicating that this fluorescence sensor can be used for the detection of thrombin content in human serum.

## 1. Introduction

As an important physiological protease, thrombin is involved in many physiological and pathological activities, such as blood coagulation, thrombosis and haemostasis [1,2]. The concentration of thrombin varies between the nM and mM levels. When a coagulation and anticoagulation imbalance occurs due to injury of the body or disease, the in vivo thrombin activity changes. Thus, quantitative detection of thrombin content for the assessment ofcoagulation ability has important significance in clinical medicine and disease diagnosis [3,4,5].

The binding between target molecules of nucleic acid aptamers and proteins selected using the technique of systematic evolution of ligands by exponential enrichment (SELEX) is similar to the binding of antibody and antigen. Nucleic acid aptamers can tightly bind to the target molecule, showing advantages of good storage attribute, high specificity, strong affinity, and high selectivity, and have been widely applied in studies to identify the aptamer sensor of different disease-related protein molecules [6,7,8,9,10,11]. Current photochemical methods for the detection of thrombin content based on aptamers include fluorescence methods [12,13], colorimetric methods [14,15], and Raman scattering [16,17], electrochemical methods include impedance methods [18,19,20,21], and polarography [22,23,24]. Kopelman’s group recently reported a new method for the detection of thrombin by magnetic bead rotation [25]. The research group of Gao reported a new thrombin detection method based on a phosphorescence energy transfer system with a detection limit of 0.013 nM [26].

In recent years, research on semiconducting polymer dots (Pdots) has received increasing attention from scholars [27,28,29,30]. Pdots have excellent optical properties, such as a large optical absorption cross section and high fluorescence quantum yield, and show good biocompatibility and colloidal stability. Pdots are therefore particularly suitable for the design of nanofluorescent probes with high fluorescent brightness and high stability [31,32,33,34].

In this study, carboxyl-functionalized Pdots synthesized by nanoprecipitation were used as the energy donors, and BHQ-labelled thrombin aptamers were used as the energy acceptors. The π–π stacking effect of the aptamers and Pdotsin close proximity produced a resonant energy transfer system, resulting in fluorescence quenching of the Pdots.

**Figure 1:**
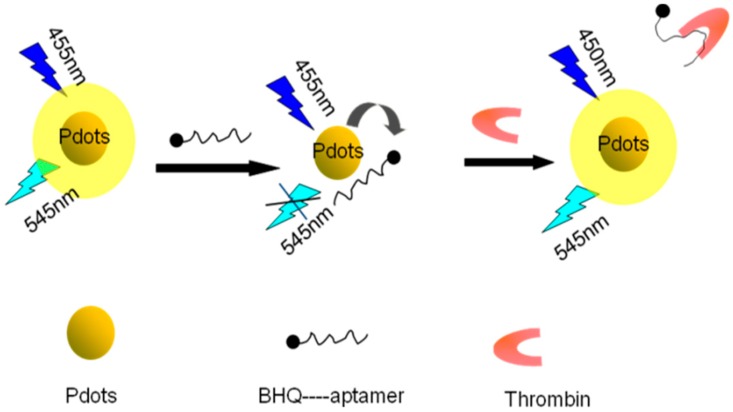
Schematic diagram of the detection of thrombin by high-efficiency energy transfer between functionalized Pdots and black hole quenching dye (BHQ).

Afterthe addition of thrombin, the strong binding of the aptamers to thrombin caused the fluorescence group to move away from the BHQ-thrombin complex, restoring the fluorescence intensity and enabling quantitative determination of thrombin (Figure 1).

## 2. Experimental Section

### 2.1. Reagents and Materials

The experimental equipment in this study includeda UV-vis UV-3010 spectrophotometer (Hitachi, Tokyo, Japan), a digital rotary evaporator (IKA, Staufen im Breisgau, Germany), an ultrasonic cleaner (Branson Ultrasonics, Danbury, CT, USA), an LS-55 fluorescence spectrophotometer (PerkinElmer, Akron, OH, USA), a quartz cuvette (1 cm × 1 cm), a laser dynamic light scattering (DLS) detector, a thermostatic shaker (Shiping, Shanghai, China), a pHS-3C pH meter (Weiye, Shanghai, China), an H-600 transmission electron microscope (Hitachi, Tokyo, Japan), an analytical balance (Yueping, Shanghai, China), a thermostatic magnetic stirrer (IKA, Staufen im Breisgau, Germany), syringe filters (Sangon Biotech, Shanghai, China), and syringes (Sangon Biotech, Shanghai, China). Luminescence lifetime were carried out on an FLS920 spectrofluorometer (Edinburgh Instruments, Livingston, UK).

The experimental materials in this study included poly[9,9-dioctylfluorenyl-2,7-diyl)-co-1,4-benzo-{2,10-3}-thiadiazole)] (PFBT) alternating copolymer and poly[styrene-co-(maleic anhydride)] (PSMA) purchased from ADS, USA, 4-(2-hydroxyethyl)-1-piperazine ethanesulphonic acid (Hepes, Sangon Biotech, Shanghai, China),tetrahydrofuran (THF 99.9%, Sigma-Aldrich, Saint Louis, MO, USA), ultrapure water and BHQ-labelled thrombin aptamer (5′-GGTTGGTGTGGTTGG-3′) (Sangon Biotech Co., Ltd., Shanghai, China).

### 2.2. Preparation of Semiconducting Polymer Quantum Dots (Pdots)

First, 250 μL of PFBT solution and 50 μL of PSMA solution (stock solutions with a mass concentration of 1 mg/mL) were used to prepare a 5 mL solution in THF, which was mixed thoroughly by ultrasonic vibration. Under ultrasonic vibration, 10 mL of ultra-pure water was quickly added to the solution, and the ultrasonic vibration was continued for 5–6 min. The resulting mixture was heated in a rotary evaporator to remove THF. After filtration and volume adjustment, a Pdots solution of 50 mg/mL with good water dispersibility was obtained. The preparation was stored in a 4 °C refrigerator until use [35,36,37].

### 2.3. Interaction between Carboxyl-Functionalized Pdots and BHQ-TBA

For the reaction, 10 μL of 50 mg/mL Pdots and different amounts of BHQ-TBA were mixed, and the volume was adjusted to 1 mL with HEPES buffer (20 mM, pH 7.4, 140 mM NaCl), followed by culturing in a thermostatic shaker for 1 h (200 r/min, 37 °C). The fluorescence intensity was measured at an excitation wavelength of 455 nm.

### 2.4. Detection of Thrombin

In a colorimetric cuvette containing 10 μL of 50 mg/mL Pdots and an optimal concentration of BHQ-TBA, different concentrations of thrombin were added, and the volume was adjusted to 1 mL with HEPES buffer, followed by reaction under the same conditions for 1 h. The fluorescence intensity was then measured.

### 2.5. Determination of Serum Thrombin

Serum samples (provided by Hospital of Anhui Normal University, Wuhu, China) were diluted 100-fold with HEPES buffer and subjected to thrombin detection. All other conditions remained unchanged, and the fluorescence intensity was measured.

## 3. Results and Discussion

**Figure 2:**
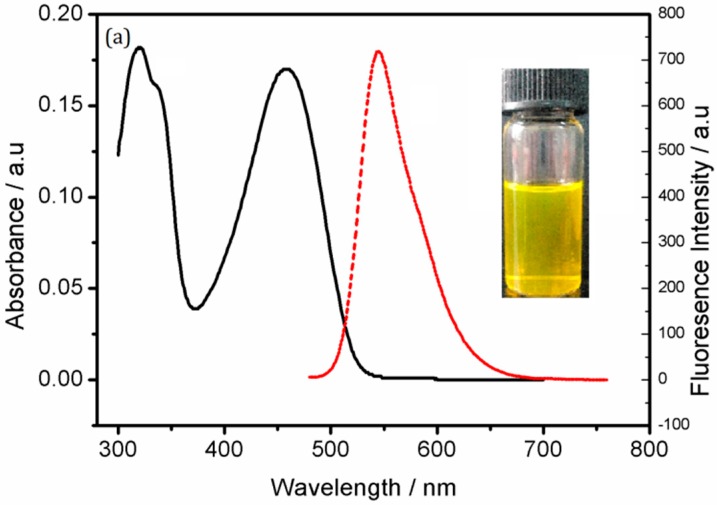

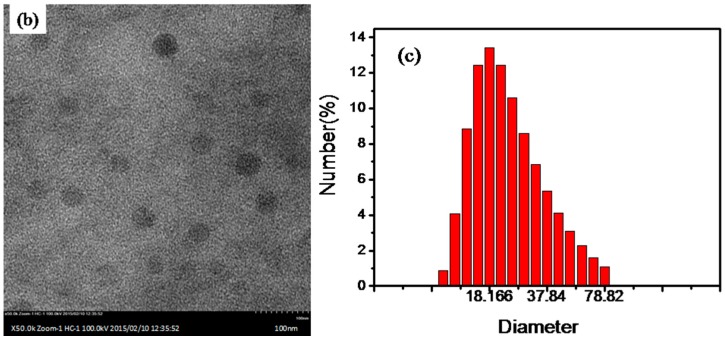
(**a**) UV-VIS absorption spectrum (solid line), fluorescence emission spectrum (dashed line, excitation at 455 nm) of Pdots in HEPES buffer (20 mM, pH 7.4). (**b**) Image of functionalized Pdots; (**c**) particle size distribution of functionalized Pdots as determined by dynamic light scattering.

A functionalized Pdots aqueous solution with good water dispersibility was prepared by the nanoprecipitation method. The UV absorption peak was observed at 455 nm by UV-VIS spectrophotometry, and the fluorescence emission peak was observed at 545 nm by fluorescence spectrophotometry (Figure 2a), consistent with findings reported in the literature [38]. On the other hand, Figure 2b shows a image of carboxyl functionalized Pdots. The functionalized Pdots were spherical particles with good water dispersibility, uniform size, a diameter of approximately 30 nm and no apparent polymerization. Figure 2c shows the particle size distribution of the functionalized Pdots by dynamic light scattering, which demonstrated that the particle size was consistent with the TEM image.

**Figure 3:**
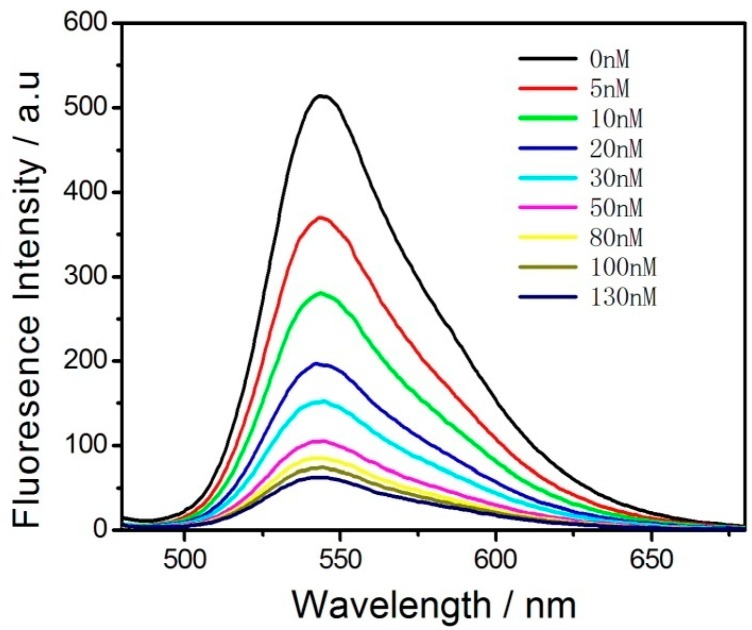
Quenching of fluorescence emission spectra of 0.5 mg/mL functionalized Pdots upon titration by BHQ-TBA in HEPES buffer (20 mM, pH 7.4). The concentrations of BHQ-TBA were 0, 5, 10, 20, 30, 50, 80, 100 and 130 nM. Excitation was performed at 455 nm.

The fluorescence spectrum of the functionalized Pdots and the absorption spectrum of BHQ-TBA greatly overlap, thus enabling energy transfer with fluorescence resonance with the functionalized Pdots as the donors and BHQ-TBA as the receptors. We investigated the degree of fluorescence quenching by adding different concentrations of BHQ-TBA to the functionalized Pdots (Figure 3). Different concentrations of BHQ-TBA solution (from 0.0 to 130 nM) were added to a solution of 0.5 mg/mL functionalized Pdots [39].

As the concentration of BHQ-TBA increased, the fluorescence intensity of the system gradually decreased. When the concentration of the BHQ-TBA solution reached 130 nM, the quenching reached a maximum. When a solution of BHQ-TBA was added to the functionalized Pdot solution, the π–π stacking effect between BHQ-TBA and the Pdots in close proximity formed a resonance energy transfer system, thereby quenching the fluorescence of the Pdots. The quenching efficiency was calculated as (1 − *F*/*F*_0_), in which *F*_0_ and *F* represent the fluorescence intensities of the functionalized Pdots in the absence (*F*_0_) and presence (*F*) of the BHQ-TBA solution (Figure S1). When 130 nM BHQ-TBA solution was added to the system, the quenching efficiency was calculated to be 88.7%, indicating that BHQ-TBA had a strong ability to quench the functionalized Pdots, laying the foundation for the design of a sensitive “turn-on” mode sensor. Meanwhile, the impact of BHQ-TBA reaction time on the fluorescence intensity of the functionalized Pdots solution is shown in Figure S2. The fluorescence intensity reached a minimal value in approximately 50 min. To ensure the stability of the fluorescence signal, the optimal incubation time was set at 60 min. To further confirm the FRET mechanism between Pdots and BHQ-TBA, time-resolved fluorescence measurements were performed by collecting the emission intensities at 545 nm. Figure S5 shows the fluorescence lifetime is estimated to be 1.09 ns for Pdots-BHQ-TBA and 0.97 ns for Pdots, respectively.

**Figure 4:**
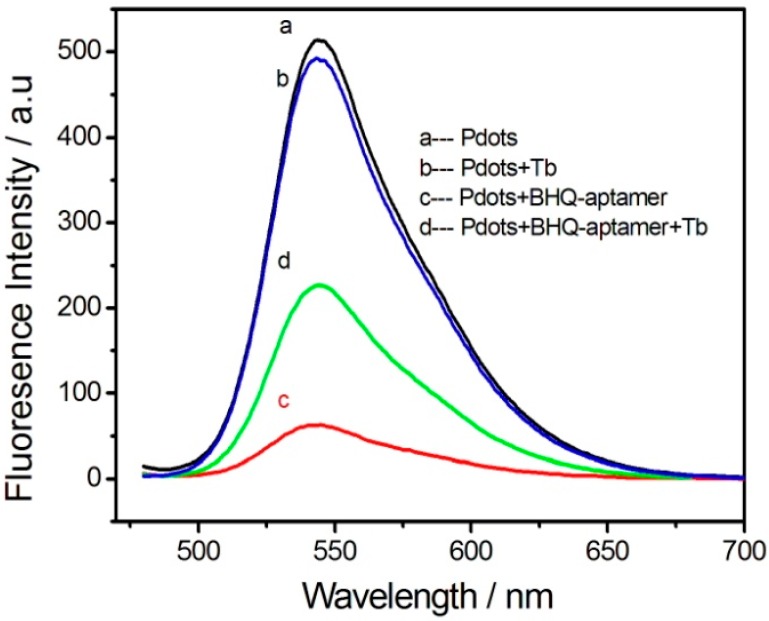
Fluorescence emission spectra of functionalized Pdots in HEPES buffer (20 mM, pH 7.4) upon addition of BHQ-TBA, thrombin. (**a**) 0.5 mg/mL functionalized Pdots; (**b**) 0.5 mg/mL functionalized Pdots after adding 20 nmol/L thrombin; (**c**) 0.5 mg/mL functionalized Pdots after adding 130 nM BHQ-TBA; (**d**) The functionalized Pdots-BHQ-TBA system after adding 20 nM thrombin in HEPES buffer. Excitation was performed at 455 nm.

The response of the functionalized Pdots-BHQ-TBA fluorescence energy transfer system to thrombin was investigated. As shown in Figure 4, Upon 20 nM thrombin was added to 0.5 mg/mL functionalized Pdots, the fluorescence of the system did not change significantly (curve b). With the addition of 130 nM BHQ-TBA solution, the fluorescence decreased to a minimum (curve c), and the addition of 20 nM thrombin to this system partially restored the fluorescence intensity of the system (curve d). The results demonstrated that after adding thrombin to the system, the ability to form the G-quadruplex structure by specific binding between BHQ-TBA and thrombin was much greater than the π–π stacking effect of BHQ-TBA and functionalized Pdots. Thus, the energy receptor BHQ-TBA bound thrombin and was released from the surface of the energy donor, resulting in the recovery of the fluorescence intensity of the energy donor. To ensure the result and obtain stable signal, a series of seven duplicate measurements was used for estimating the precision, the average of a number of measurements was chosen.

**Figure 5:**
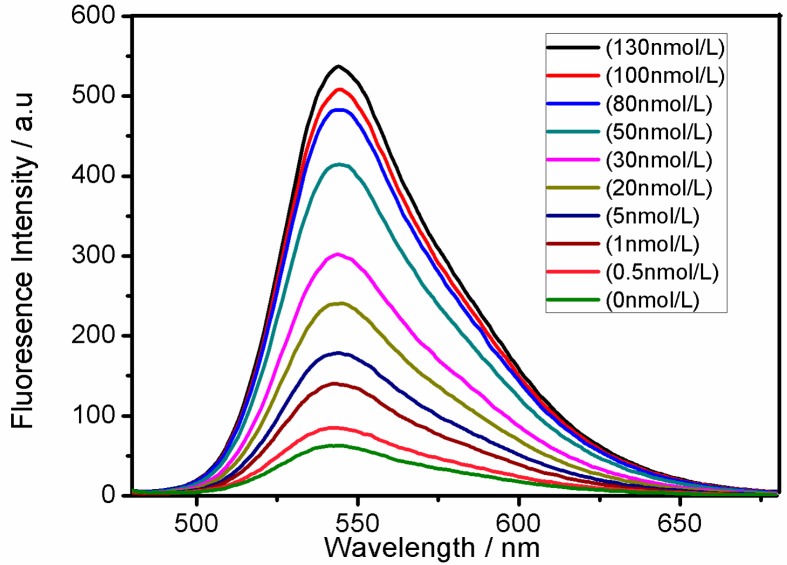
Changing of fluorescence emission spectra of thefunctionalized Pdots-BHQ-TBA system upon titration by thrombin: 0, 0.5, 1, 5, 20, 30, 50, 80, 100, and 130 nM in HEPES buffer (20 mM, pH 7.4). Excitation was performed at 455 nm.

Based on this principle of fluorescence recovery, a new method was established to detect the content of thrombin using “turn-on” fluorescence energy transfer. The recovery of fluorescence intensity when different concentrations of thrombin were added to the Pdots-BHQ-TBA system under the optimized experimental conditions is shown in Figure 5. To ensure the result and obtain stable signal, a series of seven duplicate measurements was used for estimating the precision, the average of a number of measurements was chosen.

As the thrombin concentration increased, the fluorescence intensity of the system gradually recovered. When the concentration of thrombin was 130 nM, the fluorescence of the system was recovered to the maximum, and increasing the concentration of thrombin did not increase the fluorescence of the system. Based on the definition of (*F* − *F*_0_)/*F*_0_ (*F* refers to the fluorescence intensity after adding different concentrations of thrombin, and *F*_0_ refers to the fluorescence intensity with no addition of thrombin), Figure S3 shows the linear relationship between the fluorescence recovery (*F* − *F*_0_)/*F*_0_ and the thrombin concentration. When the concentration of thrombin was in the range of 0–50 nM, a good linear relationship was observed (R^2^ = 0.990). The equation of the standard curve was (*F* − *F*_0_)/*F*_0_ = 0.7899 + 0.0621c (c is in nM). The detection limit was 0.33 nM (S/N = 3). Based on the detection for 20 nM thrombin and the standard deviation of seven repeat measurements, the relative standard deviation of this method was 4.01%, indicating that the detection of thrombin by fluorescence energy transfer in the Pdots-BHQ-TBA system was reproducible. A comparison with other methods is shown in Table 1.

**Figure 6:**
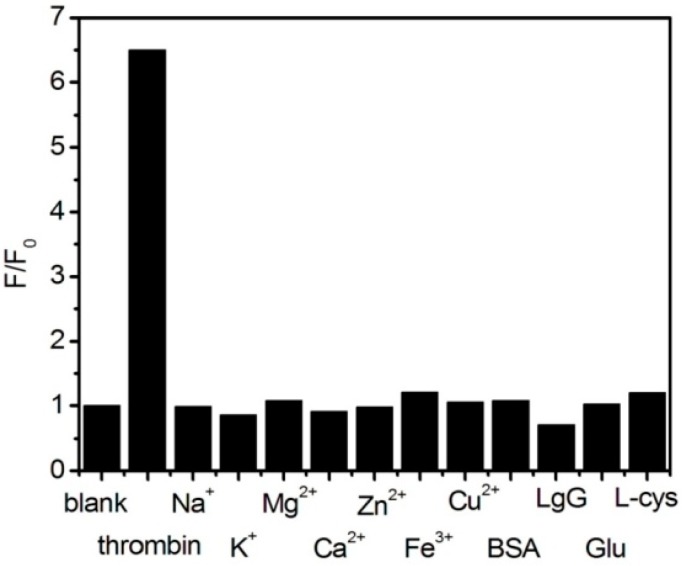
Fluorescence recovery of different substances in HEPES buffer (20 mM, pH 7.4). The concentration of all substances was 1 μM. F_0_ is fluorescence intensity at 545 nm for Pdots-BHQ-TBA, and F is the fluorescent intensity after adding different substances. Excitation was performed at 455 nm.

The impacts of the corresponding ions and similar proteins on the sensor under the same experimental conditions were investigated. Figure 6 shows the fluorescence recovery of the different substances, represented by *F/F*_0_. Except for 100 nM BSA and 130 nM thrombin, all substances were used at a concentration of 1 μM. Specific concentrations of the interfering substances were added to the Pdots-BHQ-aptamer system. As shown in Figure 6, the relative fluorescence intensity of the sensor after adding 130 nM thrombin solution was *F/F*_0_ = 6.5 (*F*_0_ refers to the fluorescence intensity of the Pdots-BHQ-aptamer system without adding any substance, and *F* refers to the fluorescence intensity of the system after adding thrombin and the relevant interfering substance). In contrast to the addition of interfering substances, the addition of thrombin solution significantly restored the fluorescence of the system. The impact of other interfering substances was negligible, and thrombin was specifically detected, confirming that thrombin solution can be detected with the “turn-on” fluorescence method.

**Figure 7:**
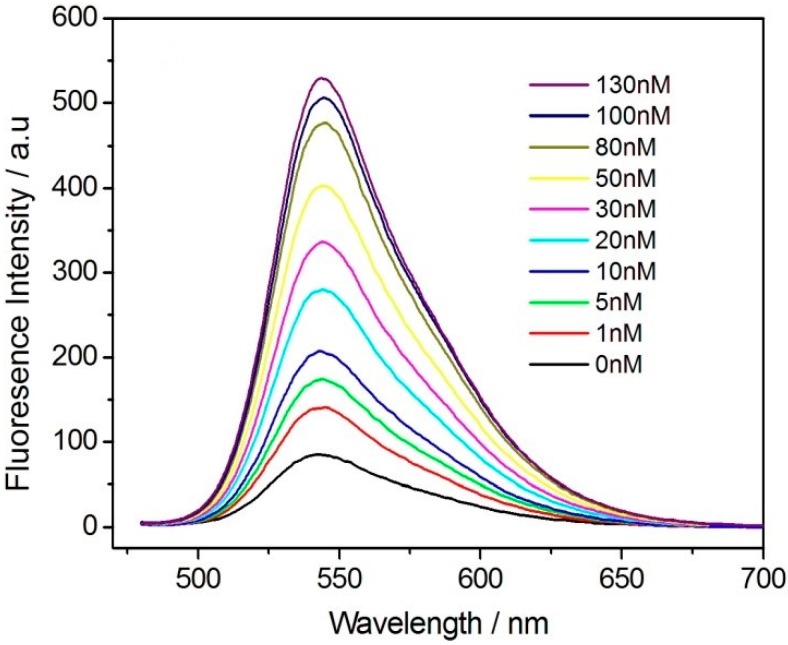
Changing of fluorescence emission spectra of the functionalized Pdots-BHQ-TBA system upon titration by thrombin to serum: 0, 1, 5, 10, 20, 30, 50, 80, 100, and 130 nM in HEPES buffer (20 mM, pH 7.4). Excitation was performed at 455 nm.

To verify the good sensitivity and specificity of this method for the detection of actual samples, the content of thrombin in serum was quantitatively detected. The serum used in the experiment was collected from healthy people. Before use, the sample to be tested was diluted 10-fold with HEPES buffer, and the concentrations of thrombin in the sample and after the addition of the standard were detected. Figure 7 shows the recovery of fluorescence intensity when thrombin at different concentrations was added to human serum under the optimized experimental conditions. The concentrations of thrombin of human serum were determined as 0.45 nM, respectively, using the calibration curve obtained in the human serum matrix, which are consistent with the reported levels [40]. As the concentration of thrombin increased, the fluorescence intensity of the system also slowly recovered. As shown in Figure S4, the linearity of the response to thrombin in the concentration range of 0–30 nM was examined, with a good linear relationship in the thrombin concentration range of 0–30 nM (R^2^ = 0.996). The linear equation was (*F/F*_0_*)/F*_0_ = 0.6933 + 0.0829c (c is in nM). Compared to the sensor in pure buffer, the sensitivity of the sensor for the detection of thrombin in serum was slightly lower. This may be the result of other factors in the serum. The detection limit was 0.58 nM (S/N = 3). Based on the detection of 5 nM thrombin and the standard deviation of seven repeat measurements, the relative standard deviation of this method was 4.7%, indicating that the detection of thrombin by fluorescence energy transfer in the Pdots-BHQ-aptamer system was reproducible. To ensure the result and obtain stable signal, a series of seven duplicate measurements was used for estimating the precision, the average of a number of measurements was chosen.

For detection in actual serum samples, thrombin at concentrations of 5.0, 10.0, and 20.0 nM were added to a 10-fold diluted serum sample, and then, the thrombin in the sample was detected by the standard addition method under optimized conditions. The different fluorescence recovery intensities obtained are shown in Table 2. Under the optimized conditions, the standard addition method was used to detect these samples. The recovery rate ranged from 96.2% to 104.1%, indicating that the fluorescence sensor can be used to detect the content of thrombin in human serum. Therefore, quantitative thrombin assay was achieved in actual serum. In one word, as a brand new analytical method, Pdots FRET has already shown its potential in bioanalytical chemistry. Moreover, there is no doubt that the analytical performances including the detection sensitivity can be further improved in future studies, through the optimization of the properties of probe as well as detection conditions.

## 4. Conclusions

In conclusion, the principle of fluorescence energy transfer was used for the sensitive quantitative detection of in vivo thrombin content, and a good response in the detection of actual samples (human serum) was confirmed. With functionalized Pdots as the energy donor and BHQ-TBA as the energy receptor, a fluorescence energy transfer system was established. Fluorescence recovery of the system was achieved by specific binding between BHQ-TBA and thrombin to form a G-quadruplex structure. A new method for the detection of thrombin by “turn-on” fluorescence energy transfer was established. The detection limit was 0.33 nM. The high fluorescent brightness and high stability of Pdots makes them a promising energy donor for FRET assay in complex biological samples, which will contribute to FRET technique as well as FRET-based analytical applications. Owing to the facile fabrication, the sensor could be readily developed to build up sensing platforms for various targets by linking different aptamers or other ligands to Pdots. Further studies looking into the energy transfer mechanism between Pdots and material would be desired to gain more comprehensive understanding and better applications.

## Figures and Tables

**Table 1:** Comparison of performances of aptamer-based sensors for thrombin detection.

Methods	Signal Output/Reporter	Linear Range (nmol/L)	Detection Limit (nmol/L)	Ref.
a DNA cycle amplified method based on ECL quenching of Fe_3_O_4_@CdSe QDs by gold NPs	ECL/Fe_3_O_4_@CdSe	0.001–5.0	0.00012	[39]
turn-On Fluorescence Sensor Based on single walled carbon nanohorn-Peptide complex	fluorescence/fluorescein	-	0.1	[40]
a fluorescence detection based on simultaneous electrostatic repulsion and π–π stacking interactions of carboxylic carbon nanoparticles with single-stranded DNA	fluorescence/fluorescein	0–120	5	[41]
a aptamer biosensor based on FRET from upconverting fluorophors to carbon nanoparticles	fluorescence/Yb,Er,NaYF_4_	0.5–20	0.18	[42]
FRET aptasensor based on the dye labeled aptamer assembled graphene	fluorescence/fluorescein amidite	0.0625–0.1875	0.0313	[43]
an electrochemical biosensor based on switching structure of aptamers from DNA/DNA duplex to DNA/target complex	current/methylene blue	6–60	3	[44]
the combination of LSPR with RRI spectrum of the gold-capped oxide nanostructure	RRI/gold-capped oxide nanostructure	0.001–100,000	1	[45]
aptamer-based turn-on fluorescent detection	fluorescence/TASPI	0–2500	50	[46]
PET-based aptamer sensor for thrombin	Phosphorescence/Mn-ZnS QDs	0–40	0.013	[26]
This study	Flurorescence/PFBT Pdots	0–50	0.33	

**Table 2:** Detection of actual samples (*n* = 6).

Sample	Spiked (nmol/L)	Found (nmol/L ± SD)	Recovery (%)
1	5.0	4.81 ± 0.02	96.2 ± 0.4
2	10.0	10.41 ± 0.03	104.1 ± 0.3
3	20.0	20.33 ± 0.04	101.7± 0.2

## Supplementary Materials

The following are available online at http://www.mdpi.com/1424-8220/18/2/589/s1.
