# Erratum: Liu, Y.-Z.; Jiang, X.-K.; Cao, W.-F.; Sun, J.-Y.; Gao, F. Detection of Thrombin Based on Fluorescence Energy Transfer between Semiconducting Polymer Dots and BHQ-Labelled Aptamers. *Sensors* 2018, *18*, 589

**DOI:** 10.3390/s18124289

**Published:** 2018-12-05

**Authors:** Yizhang Liu, Xuekai Jiang, Wenfeng Cao, Junyong Sun, Feng Gao

**Affiliations:** 1 Department of Food and Environmental Engineering, Chuzhou Vocational and Technical College, Chuzhou 239001, China; 2 Laboratory of Functionalized Molecular Solids, Ministry of Education, Anhui Key Laboratory of Chemo/Biosensing, Laboratory of Optical Probes and Bioelectrocatalysis (LOPAB), College of Chemistry and Materials Science, Anhui Normal University, Wuhu 241000, China; jiangxuekai@ahnu.edu.cn (X.J.); wfcxi17@mail.ahnu.edu.cn (W.C.); sunjy228@mail.ahnu.edu.cn (J.S.)

The authors wish to make the following corrections to their paper [1]:

The author Yizhang Liu’s affiliations were incorrect in our published paper in Sensors [1]. Therefore, they are corrected from

“(^1^ Department of Food and Environmental Engineering, Vocational and Technical College, Chuzhou 239001, China

^2^ Laboratory of Functionalized Molecular Solids, Ministry of Education, Anhui Key Laboratory of Chemo/Biosensing, Laboratory of Optical Probes and Bioelectrocatalysis (LOPAB), College of Chemistry and Materials Science, Anhui Normal University, Wuhu 241000, China)” to 

“(^1^ Department of Food and Environmental Engineering, Chuzhou Vocational and Technical College, Chuzhou 239001, China

^2^ Laboratory of Functionalized Molecular Solids, Ministry of Education, Anhui Key Laboratory of Chemo/Biosensing, Laboratory of Optical Probes and Bioelectrocatalysis (LOPAB), College of Chemistry and Materials Science, Anhui Normal University, Wuhu 241000, China)”. 

The manuscript will be updated and the original will remain online on the article webpage.

The authors would like to apologize for any inconvenience caused.
