# Supplementary material for: Detection of Thrombin Based on Fluorescence Energy Transfer between Semiconducting Polymer Dots and BHQ-Labelled Aptamers
Source: Sensors (Basel). 2018 Feb 14;18(2):589. doi: 10.3390/s18020589 (PMC5855441; doi:10.3390/s18020589)
Supplement: Supplementary file 1 [file sensors-18-00589-s001.pdf]

Supporting information

Detection of thrombin based on fluorescence energy transfer between semiconducting polymer dots and BHQ-labelled aptamers

Yizhang Liu,[*a,b] XuekaiJiang,[b] WenfengCao,[b] JunyongSun,[b] and Feng Gao[*b]

[a]Chuzhou Vocational and Technical College, Chuzhou 239001, Anhui, P. R. China

[b]Laboratory of Functionalized Molecular Solids, Ministry of Education, Anhui Key Laboratory of Chemo/Biosensing, Laboratory of Optical Probes and Bioelectrocatalysis (LOPAB), College of Chemistry and Materials Science, Anhui Normal University, Wuhu 241000, P. R. China.

*E-Mail: liuyizhang111@126.com(Y. Liu); fgao@mail.ahnu.edu.cn(F. Gao)

Fluorescence energy transfer between functionalized Pdots and BHQ-TBA.

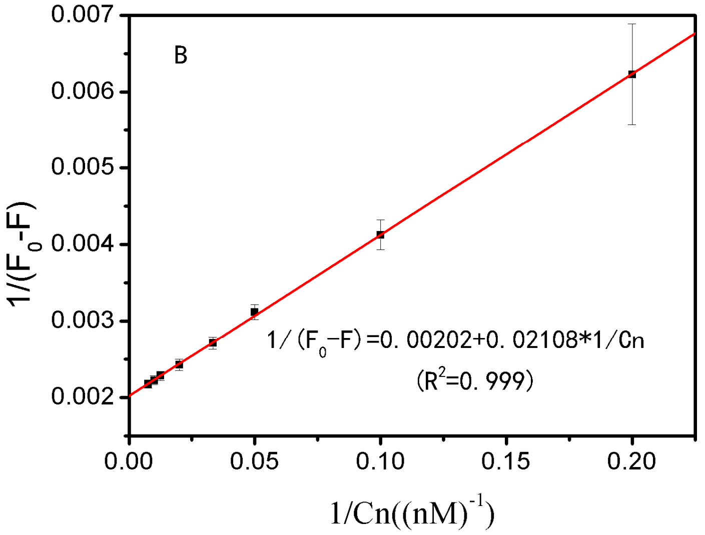

Figure S1. Bensei-Hilderbrand plot of Pdots with varied concentrations BHQ-TBA at 298 K.

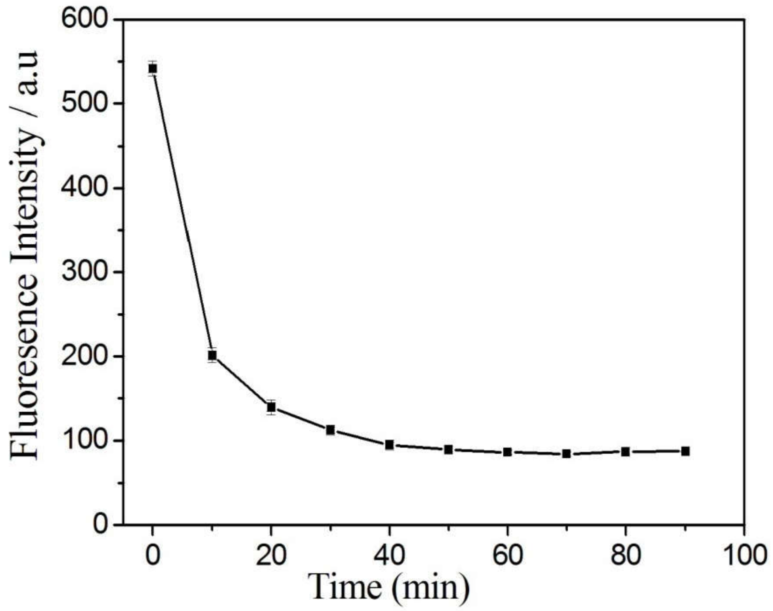

Figure S2. Impact of the reaction time on the fluorescence intensity of the system. The optimal incubation time was set at 60 minutes.

Detection of thrombin by fluorescence energy transfer in the "turn-on" mode.

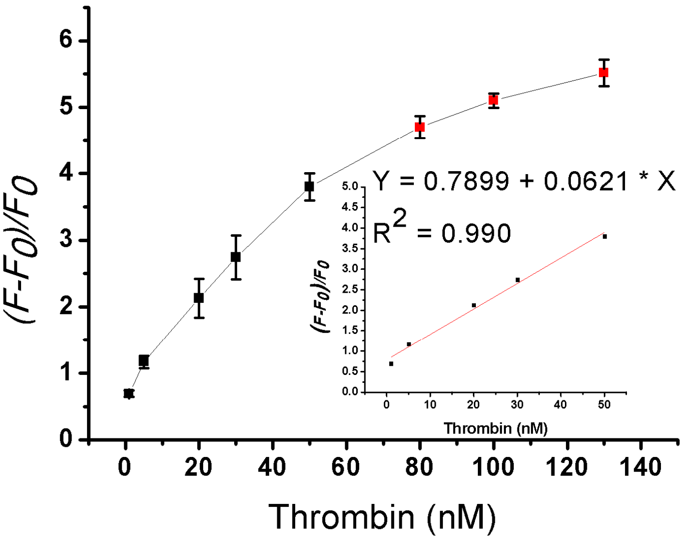

Figure S3. Plots of the fluorescence intensity ratio (F-F_0)/F as a function of thrombin concentration. The inset shows the linearity of the fluorescence intensity ratios (F-F_0)/F against the thrombin concentrations.

Detection of actual samples

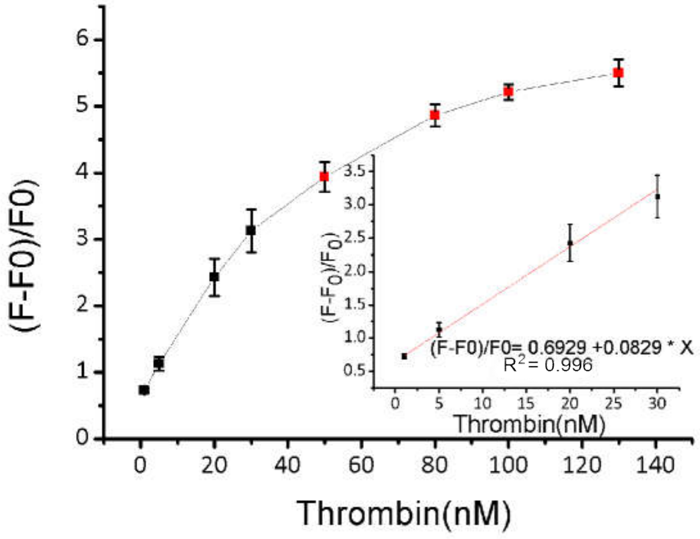

Figure S4. Plots of the fluorescence intensity ratio (F-F_0)/F as a function of thrombin concentration in serum. The inset shows the linearity of the fluorescence intensity ratios (F-F_0)/F against the thrombin concentrations in serum.

Detection of Lifetime

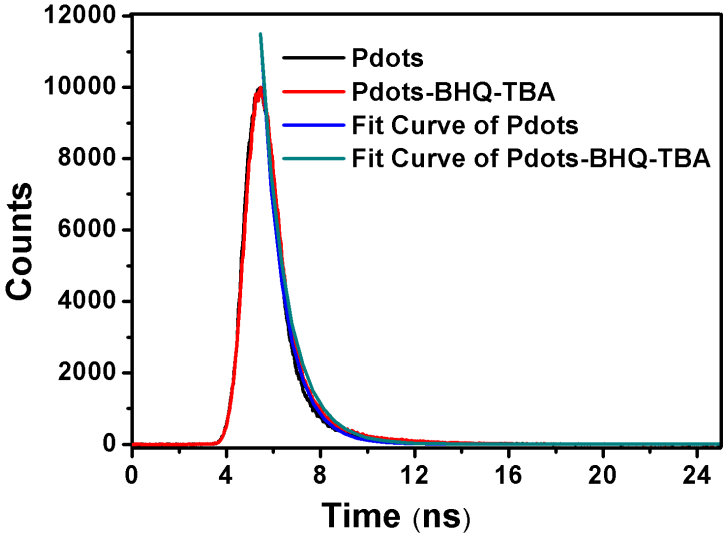

Figure S5. Time-resolved fluorescence spectra of Pdots (black dot line) and Pdots-BHQ-TBA (red dot line). Time-resolved fluorescence measurements were performed by collecting the emission intensities at 545 nm.
